# Remarkable Vitality

**Published:** 1879-10

**Authors:** William H. Slacer


### REMARKABLE VITALITY.

REPORTED BY WILLIAM H. SLACER, M. D.

Mr. J., aged 48, weighing over 200 lbs., was struck by a locomotive and thrown some fifteen feet, falling heavily on the pavement; the accident occurred about 8 A. M.; soon regaining his feet he walked home, nearly a quarter of a mile distant.

Was lying on a sofa when I saw him, which was less than one hour after injury. Examination revealed severe scalp wound on left side, about two and a half inches long; left clavicle fractured and driven downwards; infra-clavicular region much bruised; abrasion on right arm. He appeared as though fast going into collapse; pulse 120 and feeble; extremities cold, respiration thirty per minute; complained of severe pain in region of heart; conversed freely. I ordered him to be put in bed; he refused as unnecessary, but after considerable urging he consented to comply, providing I allowed him to go without assistance; said he was not hurt badly. He walked, without help, up one flight of stairs, undressed and went to bed. I soon became satisfied that he had serious internal injuries; placed him in the most comfortable position, and without making a thorough examination, I endeavored to bring about reaction; gave hypodermic injections of morphine to relieve pain, heat to extremities, stimulants, etc.

10 A. M. Vomited a little clotted blood; detect crepitation right lung; air enters both lungs freely; respiratory murmur clear in left lung.

12 M. Less pain; pulse 124, fuller; temperature 99; respiration 30; symptoms slightly improved.

3 P. M. Pulse 128; respiration 32; temperature normal; has taken some beef tea; has not vomited since 10 A. M.

6 P. M. Weaker; pulse 138; respiration 34.

10 P. M. Gradually sinking; pulse 140; respiration 38 and labored; died at midnight.

Post-mortem following day revealed, besides the external injuries already described, a comminuted fracture of left clavicle, which had penetrated the apex of left lung, first six ribs fractured about their middle, one or two were torn from the sternum, four had penetrated lung substance; on right side fourth, fifth and sixth ribs fractured near sternal end, and lung punctured in several places; lungs collapsed; thoracic cavity nearly filled with bloody serum.

During illness, there was an entire absence of brain trouble, the patient being perfectly conscious to the last moment of his life.

